# *N*′-[(*E*)-4-Hydr­oxy-3-methoxy­benzyl­idene]benzohydrazide

**DOI:** 10.1107/S1600536809044122

**Published:** 2009-10-28

**Authors:** Zahid Shafiq, Muhammad Yaqub, M. Nawaz Tahir, Abid Hussain, M. Saeed Iqbal

**Affiliations:** a Department of Chemistry, Bahauddin Zakariya University, Multan 60800, Pakistan; b Department of Physics, University of Sargodha, Sargodha, Pakistan; c Department of Chemistry, Government College University, Lahore, Pakistan

## Abstract

In the title compound, C_15_H_14_N_2_O_3_, the phenyl ring is disordered over two set of sites with an occupancy ratio of 0.810 (3):0.190 (3); the dihedral angle between the two components is 72.3 (4)°. The benzene and phenyl rings are oriented at dihedral angles of 69.18 (8) and 26.0 (5)° (major and minor orientations, respectively), and an intra­molecular O—H⋯O hydrogen bond occurs. In the crystal, mol­ecules are linked by N—H⋯O, O—H⋯O and C—H⋯O inter­actions, generating a three-dimensional network.

## Related literature

For related structures, see: Shafiq *et al.* (2009*a* ▶,*b* ▶); Shi (2005 ▶).
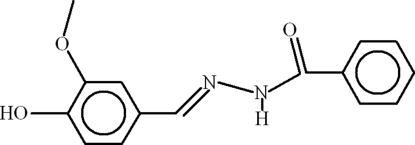


## Experimental

### 

#### Crystal data

C_15_H_14_N_2_O_3_
*M* *_r_* = 270.28
Tetragonal, 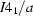

*a* = 18.6994 (8) Å
*c* = 15.7223 (12) Å
*V* = 5497.6 (5) Å^3^
*Z* = 16
Mo *K*α radiation
μ = 0.09 mm^−1^
*T* = 296 K
0.28 × 0.24 × 0.22 mm

#### Data collection

Bruker Kappa APEXII CCD diffractometer
Absorption correction: multi-scan (*SADABS*; Bruker, 2005 ▶) *T*_min_ = 0.976, *T*_max_ = 0.979
15310 measured reflections
3393 independent reflections
1656 reflections with *I* > 2σ(*I*)
*R*_int_ = 0.036

#### Refinement

*R*[*F* ^2^ > 2σ(*F* ^2^)] = 0.048
*wR*(*F* ^2^) = 0.151
*S* = 0.99
3393 reflections
214 parameters
H-atom parameters constrained
Δρ_max_ = 0.22 e Å^−3^
Δρ_min_ = −0.14 e Å^−3^

### 

Data collection: *APEX2* (Bruker, 2007 ▶); cell refinement: *SAINT* (Bruker, 2007 ▶); data reduction: *SAINT*; program(s) used to solve structure: *SHELXS97* (Sheldrick, 2008 ▶); program(s) used to refine structure: *SHELXL97* (Sheldrick, 2008 ▶); molecular graphics: *ORTEP-3* (Farrugia, 1997 ▶) and *PLATON* (Spek, 2009 ▶); software used to prepare material for publication: *WinGX* (Farrugia, 1999 ▶) and *PLATON*.

## Supplementary Material

Crystal structure: contains datablocks global, I. DOI: 10.1107/S1600536809044122/hb5175sup1.cif

Structure factors: contains datablocks I. DOI: 10.1107/S1600536809044122/hb5175Isup2.hkl

Additional supplementary materials:  crystallographic information; 3D view; checkCIF report

## Figures and Tables

**Table 1:** Hydrogen-bond geometry (Å, °)

*D*—H⋯*A*	*D*—H	H⋯*A*	*D*⋯*A*	*D*—H⋯*A*
O2—H2⋯O3	0.82	2.21	2.653 (2)	115
N1—H1⋯O1^i^	0.86	2.14	2.8633 (19)	142
O2—H2⋯O1^ii^	0.82	2.05	2.773 (2)	146
C2*A*—H2*A*⋯O1^i^	0.93	2.43	3.165 (2)	136

Symmetry codes: (i) 
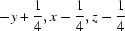
; (ii) 
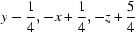
.

**Fig. 1.**
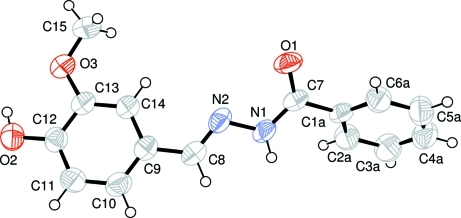
View of (I) showing the major disorder component. The displacement ellipsoids are drawn at the 50% probability level. H-atoms are shown by circles of arbitrary radius.

**Fig. 2.**
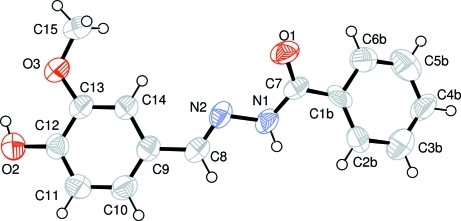
View of (I) showing the minor disorder component. The displacement ellipsoids are drawn at the 50% probability level. H-atoms are shown by circles of arbitrary radius.

**Fig. 3.**
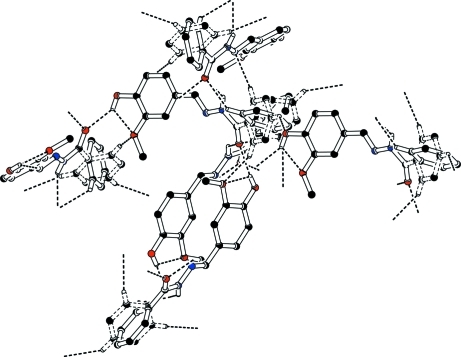
The partial packing of (I), which shows that molecules form three dimensional polymeric network. H-atoms not involved in H-bondings have been omitted for clarity.

## supplementary crystallographic information

### Comment

Recently we have reported the crystal structures of (II) *N*'-[(*E*)-(4-Bromo-2-thienyl)methylene]isonicotinohydrazide (Shafiq *et al.*, 2009*a*) and (III) *N*'-[(*E*)-(4-Bromo-2-thienyl)methylidene]benzohydrazide 0.06-hydrate (Shafiq *et al.*, 2009*b*). The title compound (I, Fig. 1), has been prepared in continuation of synthesizing various hydrazide derivatives.

The crystal structure of (IV) (*E*)-*N*-Benzoyl-*N*'-(3-hydroxy-4-methoxybenzylidene)hydrazine (Shi, 2005) has been published which differs from (I) due to positional change of hydroxy and methoxy.

In the title compound benzene ring of benzohydrazide is disordered over two set of sites with occupancy ratio of 0.810 (3):0.190 (3). The majority and miniority groups A (C1A—C6A) and B (C1B—C6B) respectively, are oriented at a dihedral angle of 72.27 (36)°. The benzene ring C (C9—C14) of 4-Hydroxy-3-methoxyphenyl is of course planar. The dihedral angle between A/C and B/C is 69.18 (8)° and 25.98 (51)°, respectively. The molecules are stabilized in the form of three dimensional polymeric network due to strong intra as well as intermolecular H-bondings (Table 1, Fig.2).

### Experimental

To a hot stirred solution of benzoic hydrazide (1.36 g, 0.01 mol) in ethanol (15 ml) was added vanillin (1.52 g, 0.01 mol). The resultant mixture was then heated under reflux. After an hour precipitates were formed. The reaction mixture was further heated about 30 min for the completion of the reaction which was monitored through TLC. The reaction mixture was cooled to room temperature, filtered and washed with hot ethanol.  Colourless prisms of (I) were obtained by recrystallization of the crude product in 1,4-dioxan:ethanol (1:1) after four days.

### Refinement

The disordered phenyl rings A (C1A—C6A) and B (C1B—C6B) were refined using AFIX 66 and all atoms have independent anisotropic thermal parameters.

The H-atoms were positioned geometrically (O–H = 0.82 Å, N–H = 0.86 Å, C–H = 0.93–0.96 Å) and refined as riding with *U*_iso_(H) = 1.2*U*_eq_(carrier) or 1.5*U*_eq_(methyl C).

### Crystal data

**Table:**

C_15_H_14_N_2_O_3_	*D*_x_ = 1.306 Mg m^−^^3^
*M**_r_* = 270.28	Mo *K*α radiation, λ = 0.71073 Å
Tetragonal, *I*4_1_/*a*	Cell parameters from 3393 reflections
Hall symbol: -I 4ad	θ = 1.7–28.3°
*a* = 18.6994 (8) Å	µ = 0.09 mm^−^^1^
*c* = 15.7223 (12) Å	*T* = 296 K
*V* = 5497.6 (5) Å^3^	Prism, colourless
*Z* = 16	0.28 × 0.24 × 0.22 mm
*F*(000) = 2272	

### Data collection

**Table:**

Bruker Kappa APEXII CCD diffractometer	3393 independent reflections
Radiation source: fine-focus sealed tube	1656 reflections with *I* > 2σ(*I*)
graphite	*R*_int_ = 0.036
Detector resolution: 7.40 pixels mm^-1^	θ_max_ = 28.3°, θ_min_ = 1.7°
ω scans	*h* = −24→24
Absorption correction: multi-scan (*SADABS*; Bruker, 2005)	*k* = −24→24
*T*_min_ = 0.976, *T*_max_ = 0.979	*l* = −20→20
15310 measured reflections	

### Refinement

**Table:**

Refinement on *F*^2^	Primary atom site location: structure-invariant direct methods
Least-squares matrix: full	Secondary atom site location: difference Fourier map
*R*[*F*^2^ > 2σ(*F*^2^)] = 0.048	Hydrogen site location: inferred from neighbouring sites
*wR*(*F*^2^) = 0.151	H-atom parameters constrained
*S* = 0.99	*w* = 1/[σ^2^(*F*_o_^2^) + (0.071*P*)^2^ + 0.721*P*] where *P* = (*F*_o_^2^ + 2*F*_c_^2^)/3
3393 reflections	(Δ/σ)_max_ < 0.001
214 parameters	Δρ_max_ = 0.22 e Å^−^^3^
0 restraints	Δρ_min_ = −0.14 e Å^−^^3^

### Special details

**Table:**

Geometry. Bond distances, angles *etc*. have been calculated using the rounded fractional coordinates. All su's are estimated from the variances of the (full) variance-covariance matrix. The cell e.s.d.'s are taken into account in the estimation of distances, angles and torsion angles
Refinement. Refinement of *F*^2^ against ALL reflections. The weighted *R*-factor *wR* and goodness of fit *S* are based on *F*^2^, conventional *R*-factors *R* are based on *F*, with *F* set to zero for negative *F*^2^. The threshold expression of *F*^2^ > σ(*F*^2^) is used only for calculating *R*-factors(gt) *etc*. and is not relevant to the choice of reflections for refinement. *R*-factors based on *F*^2^ are statistically about twice as large as those based on *F*, and *R*- factors based on ALL data will be even larger.

### Fractional atomic coordinates and isotropic or equivalent isotropic displacement parameters (Å^2^)

**Table:**

	*x*	*y*	*z*	*U*_iso_*/*U*_eq_	Occ. (<1)
O1	0.31015 (8)	0.09452 (8)	0.50043 (7)	0.0639 (5)	
O2	−0.07897 (8)	−0.13841 (8)	0.63057 (9)	0.0715 (6)	
O3	−0.00552 (9)	−0.02825 (9)	0.69117 (8)	0.0751 (6)	
N1	0.23647 (9)	0.04528 (9)	0.40397 (9)	0.0577 (6)	
N2	0.19267 (9)	0.01533 (10)	0.46558 (9)	0.0578 (6)	
C1A	0.32949 (10)	0.12512 (10)	0.35611 (10)	0.0491 (15)	0.813 (3)
C2A	0.29168 (9)	0.15525 (12)	0.28897 (12)	0.0678 (10)	0.813 (3)
C3A	0.32797 (12)	0.19079 (13)	0.22442 (11)	0.0813 (15)	0.813 (3)
C4A	0.40207 (12)	0.19620 (12)	0.22701 (11)	0.0810 (16)	0.813 (3)
C5A	0.43989 (9)	0.16606 (11)	0.29416 (13)	0.0760 (12)	0.813 (3)
C6A	0.40360 (10)	0.13052 (10)	0.35871 (10)	0.0619 (10)	0.813 (3)
C7	0.29070 (11)	0.08778 (10)	0.42620 (10)	0.0495 (7)	
C8	0.14721 (12)	−0.02955 (12)	0.43724 (12)	0.0584 (7)	
C9	0.09173 (11)	−0.06060 (11)	0.49065 (11)	0.0550 (7)	
C10	0.05165 (12)	−0.11712 (12)	0.46193 (12)	0.0638 (8)	
C11	−0.00483 (12)	−0.14403 (11)	0.51003 (13)	0.0636 (8)	
C12	−0.02232 (11)	−0.11260 (11)	0.58606 (12)	0.0559 (7)	
C13	0.01804 (11)	−0.05554 (11)	0.61574 (11)	0.0564 (7)	
C14	0.07470 (11)	−0.02999 (11)	0.56916 (11)	0.0576 (7)	
C15	0.03358 (16)	0.02801 (17)	0.72880 (14)	0.0990 (11)	
C2B	0.3203 (5)	0.1020 (4)	0.2723 (5)	0.059 (4)	0.187 (3)
C3B	0.3506 (6)	0.1424 (5)	0.2075 (4)	0.076 (5)	0.187 (3)
C4B	0.3772 (7)	0.2103 (5)	0.2246 (5)	0.078 (7)	0.187 (3)
C5B	0.3737 (6)	0.2377 (4)	0.3067 (6)	0.092 (6)	0.187 (3)
C6B	0.3434 (5)	0.1973 (4)	0.3716 (4)	0.070 (5)	0.187 (3)
C1B	0.3167 (5)	0.1294 (4)	0.3544 (4)	0.055 (7)	0.187 (3)
H5A	0.48947	0.16967	0.29590	0.0913*	0.813 (3)
H6A	0.42890	0.11036	0.40363	0.0743*	0.813 (3)
H10	0.06233	−0.13774	0.40962	0.0766*	
H11	−0.03064	−0.18328	0.49061	0.0763*	
H14	0.10184	0.00784	0.58997	0.0691*	
H15A	0.03617	0.06754	0.68996	0.1486*	
H15B	0.08102	0.01179	0.74192	0.1486*	
H15C	0.01014	0.04298	0.78013	0.1486*	
H8	0.14959	−0.04294	0.38034	0.0701*	
H1	0.22866	0.03642	0.35111	0.0692*	
H2	−0.08451	−0.11459	0.67387	0.1072*	
H2A	0.24210	0.15164	0.28723	0.0814*	0.813 (3)
H3A	0.30267	0.21096	0.17949	0.0978*	0.813 (3)
H4A	0.42635	0.21997	0.18383	0.0970*	0.813 (3)
H2B	0.30242	0.05661	0.26082	0.0709*	0.187 (3)
H3B	0.35296	0.12407	0.15254	0.0912*	0.187 (3)
H4B	0.39750	0.23733	0.18123	0.0932*	0.187 (3)
H5B	0.39151	0.28312	0.31819	0.1097*	0.187 (3)
H6B	0.34098	0.21565	0.42646	0.0839*	0.187 (3)

### Atomic displacement parameters (Å^2^)

**Table:**

	*U*^11^	*U*^22^	*U*^33^	*U*^12^	*U*^13^	*U*^23^
O1	0.0871 (11)	0.0700 (10)	0.0346 (6)	−0.0024 (8)	−0.0072 (6)	−0.0015 (6)
O2	0.0768 (11)	0.0677 (11)	0.0699 (9)	−0.0116 (8)	0.0145 (8)	0.0004 (7)
O3	0.0816 (11)	0.0916 (12)	0.0521 (8)	−0.0187 (9)	0.0146 (7)	−0.0128 (7)
N1	0.0632 (11)	0.0790 (12)	0.0309 (7)	−0.0057 (9)	0.0011 (7)	0.0057 (7)
N2	0.0605 (11)	0.0739 (12)	0.0390 (8)	0.0006 (10)	0.0052 (7)	0.0098 (8)
C1A	0.064 (3)	0.050 (3)	0.0334 (19)	0.0030 (19)	−0.0007 (14)	−0.0007 (18)
C2A	0.0692 (19)	0.083 (2)	0.0512 (14)	−0.0108 (16)	−0.0127 (13)	0.0175 (14)
C3A	0.099 (3)	0.092 (3)	0.0528 (16)	−0.015 (2)	−0.0081 (18)	0.0242 (17)
C4A	0.105 (3)	0.068 (3)	0.070 (2)	−0.017 (2)	0.0224 (19)	−0.0005 (19)
C5A	0.064 (2)	0.077 (2)	0.087 (2)	−0.0071 (16)	0.0126 (16)	−0.0008 (16)
C6A	0.0558 (19)	0.0681 (19)	0.0618 (15)	0.0009 (13)	−0.0030 (12)	0.0011 (12)
C7	0.0602 (13)	0.0538 (12)	0.0346 (9)	0.0093 (10)	−0.0013 (8)	0.0004 (8)
C8	0.0648 (14)	0.0671 (14)	0.0433 (10)	0.0071 (11)	0.0044 (9)	0.0017 (9)
C9	0.0589 (13)	0.0566 (13)	0.0494 (10)	0.0046 (10)	0.0034 (9)	0.0041 (9)
C10	0.0740 (15)	0.0623 (15)	0.0552 (11)	0.0051 (12)	0.0086 (11)	−0.0063 (10)
C11	0.0727 (16)	0.0500 (13)	0.0680 (13)	−0.0021 (11)	0.0014 (11)	−0.0046 (10)
C12	0.0618 (14)	0.0516 (13)	0.0544 (11)	0.0014 (10)	0.0027 (10)	0.0070 (9)
C13	0.0640 (14)	0.0615 (14)	0.0437 (10)	0.0030 (11)	0.0025 (9)	0.0010 (9)
C14	0.0619 (14)	0.0634 (14)	0.0474 (10)	−0.0053 (10)	0.0008 (9)	0.0013 (9)
C15	0.110 (2)	0.127 (2)	0.0599 (14)	−0.0388 (18)	0.0150 (13)	−0.0343 (14)
C2B	0.053 (7)	0.058 (8)	0.067 (7)	−0.013 (6)	0.004 (5)	0.002 (6)
C3B	0.081 (10)	0.089 (11)	0.058 (7)	0.012 (9)	0.008 (6)	0.007 (7)
C4B	0.098 (15)	0.084 (14)	0.051 (9)	−0.001 (11)	0.026 (8)	0.008 (8)
C5B	0.094 (11)	0.063 (9)	0.118 (12)	−0.014 (8)	−0.003 (8)	−0.001 (8)
C6B	0.084 (9)	0.066 (9)	0.060 (6)	−0.011 (7)	0.000 (6)	−0.005 (6)
C1B	0.029 (7)	0.066 (15)	0.071 (14)	−0.016 (7)	−0.013 (7)	−0.013 (11)

### Geometric parameters (Å, °)

**Table:**

O1—C7	1.229 (2)	C9—C14	1.397 (3)
O2—C12	1.358 (2)	C9—C10	1.372 (3)
O3—C13	1.364 (2)	C10—C11	1.393 (3)
O3—C15	1.411 (3)	C11—C12	1.372 (3)
O2—H2	0.8200	C12—C13	1.388 (3)
N1—N2	1.387 (2)	C13—C14	1.374 (3)
N1—C7	1.335 (3)	C2A—H2A	0.9300
N2—C8	1.275 (3)	C2B—H2B	0.9300
N1—H1	0.8600	C3A—H3A	0.9300
C1A—C6A	1.390 (3)	C3B—H3B	0.9300
C1A—C7	1.493 (2)	C4A—H4A	0.9300
C1A—C2A	1.390 (3)	C4B—H4B	0.9300
C1B—C2B	1.390 (10)	C5A—H5A	0.9300
C1B—C6B	1.391 (11)	C5B—H5B	0.9300
C1B—C7	1.455 (7)	C6A—H6A	0.9300
C2A—C3A	1.390 (3)	C6B—H6B	0.9300
C2B—C3B	1.389 (12)	C8—H8	0.9300
C3A—C4A	1.390 (3)	C10—H10	0.9300
C3B—C4B	1.390 (14)	C11—H11	0.9300
C4A—C5A	1.390 (3)	C14—H14	0.9300
C4B—C5B	1.390 (12)	C15—H15B	0.9600
C5A—C6A	1.390 (3)	C15—H15C	0.9600
C5B—C6B	1.390 (12)	C15—H15A	0.9600
C8—C9	1.456 (3)		
			
C13—O3—C15	118.43 (18)	C12—C13—C14	120.50 (17)
C12—O2—H2	109.00	O3—C13—C12	113.85 (18)
N2—N1—C7	120.41 (14)	C9—C14—C13	120.28 (19)
N1—N2—C8	114.55 (15)	C1A—C2A—H2A	120.00
N2—N1—H1	120.00	C3A—C2A—H2A	120.00
C7—N1—H1	120.00	C1B—C2B—H2B	120.00
C2A—C1A—C6A	120.00 (16)	C3B—C2B—H2B	120.00
C2A—C1A—C7	120.21 (17)	C4A—C3A—H3A	120.00
C6A—C1A—C7	119.78 (15)	C2A—C3A—H3A	120.00
C2B—C1B—C6B	120.0 (7)	C4B—C3B—H3B	120.00
C2B—C1B—C7	122.6 (6)	C2B—C3B—H3B	120.00
C6B—C1B—C7	117.2 (5)	C3A—C4A—H4A	120.00
C1A—C2A—C3A	120.00 (17)	C5A—C4A—H4A	120.00
C1B—C2B—C3B	120.0 (7)	C5B—C4B—H4B	120.00
C2A—C3A—C4A	120.01 (18)	C3B—C4B—H4B	120.00
C2B—C3B—C4B	120.1 (7)	C6A—C5A—H5A	120.00
C3A—C4A—C5A	120.00 (18)	C4A—C5A—H5A	120.00
C3B—C4B—C5B	120.0 (8)	C4B—C5B—H5B	120.00
C4A—C5A—C6A	120.00 (17)	C6B—C5B—H5B	120.00
C4B—C5B—C6B	120.0 (8)	C1A—C6A—H6A	120.00
C1A—C6A—C5A	120.00 (16)	C5A—C6A—H6A	120.00
C1B—C6B—C5B	120.0 (6)	C5B—C6B—H6B	120.00
O1—C7—C1A	120.62 (18)	C1B—C6B—H6B	120.00
O1—C7—C1B	125.7 (3)	C9—C8—H8	119.00
O1—C7—N1	122.31 (17)	N2—C8—H8	119.00
N1—C7—C1A	117.02 (14)	C9—C10—H10	120.00
N1—C7—C1B	111.7 (3)	C11—C10—H10	120.00
N2—C8—C9	122.43 (17)	C12—C11—H11	120.00
C8—C9—C10	120.45 (17)	C10—C11—H11	120.00
C8—C9—C14	120.57 (18)	C13—C14—H14	120.00
C10—C9—C14	118.80 (18)	C9—C14—H14	120.00
C9—C10—C11	120.91 (18)	O3—C15—H15C	109.00
C10—C11—C12	119.94 (19)	O3—C15—H15B	109.00
C11—C12—C13	119.53 (19)	H15B—C15—H15C	109.00
O2—C12—C11	118.86 (18)	H15A—C15—H15B	109.00
O2—C12—C13	121.61 (17)	H15A—C15—H15C	109.00
O3—C13—C14	125.62 (18)	O3—C15—H15A	109.00
			
C15—O3—C13—C12	177.4 (2)	C4A—C5A—C6A—C1A	0.0 (3)
C15—O3—C13—C14	−4.5 (3)	N2—C8—C9—C10	−169.1 (2)
C7—N1—N2—C8	−173.59 (19)	N2—C8—C9—C14	15.8 (3)
N2—N1—C7—O1	10.0 (3)	C8—C9—C10—C11	−175.5 (2)
N2—N1—C7—C1A	−172.51 (17)	C14—C9—C10—C11	−0.3 (3)
N1—N2—C8—C9	−173.17 (18)	C8—C9—C14—C13	174.08 (19)
C6A—C1A—C2A—C3A	0.0 (3)	C10—C9—C14—C13	−1.1 (3)
C7—C1A—C2A—C3A	179.15 (19)	C9—C10—C11—C12	1.9 (3)
C2A—C1A—C6A—C5A	0.0 (3)	C10—C11—C12—O2	178.01 (19)
C7—C1A—C6A—C5A	−179.15 (17)	C10—C11—C12—C13	−2.1 (3)
C2A—C1A—C7—O1	−140.5 (2)	O2—C12—C13—O3	−1.2 (3)
C2A—C1A—C7—N1	42.0 (3)	O2—C12—C13—C14	−179.40 (18)
C6A—C1A—C7—O1	38.6 (3)	C11—C12—C13—O3	178.91 (18)
C6A—C1A—C7—N1	−138.89 (19)	C11—C12—C13—C14	0.7 (3)
C1A—C2A—C3A—C4A	0.0 (3)	O3—C13—C14—C9	−177.09 (19)
C2A—C3A—C4A—C5A	0.0 (3)	C12—C13—C14—C9	0.9 (3)
C3A—C4A—C5A—C6A	0.0 (3)		

### Hydrogen-bond geometry (Å, °)

**Table:**

*D*—H···*A*	*D*—H	H···*A*	*D*···*A*	*D*—H···*A*
O2—H2···O3	0.82	2.21	2.653 (2)	115
N1—H1···O1^i^	0.86	2.14	2.8633 (19)	142
O2—H2···O1^ii^	0.82	2.05	2.773 (2)	146
C2A—H2A···O1^i^	0.93	2.43	3.165 (2)	136

Symmetry codes: (i) −*y*+1/4, *x*−1/4, *z*−1/4; (ii) *y*−1/4, −*x*+1/4, −*z*+5/4.
